# Quorum Sensing-Regulated Phenol-Soluble Modulins Limit Persister Cell Populations in *Staphylococcus aureus*

**DOI:** 10.3389/fmicb.2018.00255

**Published:** 2018-02-20

**Authors:** Martin S. Bojer, Søren Lindemose, Martin Vestergaard, Hanne Ingmer

**Affiliations:** ^1^Faculty of Health and Medical Sciences, Department of Veterinary and Animal Sciences, University of Copenhagen, Copenhagen, Denmark; ^2^Centre for Bacterial Stress Response and Persistence, University of Copenhagen, Copenhagen, Denmark

**Keywords:** *S. aureus*, persister cells, quorum sensing, *agr*, phenol-soluble modulins, supernatant

## Abstract

Incomplete killing of bacterial pathogens by antibiotics is an underlying cause of treatment failure and accompanying complications. Among those avoiding chemotherapy are persisters being individual cells in a population that for extended periods of time survive high antibiotic concentrations proposedly by being in a quiescent state refractory to antibiotic killing. While investigating the human pathogen *Staphylococcus aureus* and the influence of growth phase on persister formation, we noted that spent supernatants of stationary phase cultures of *S. aureus* or *S. epidermidis*, but not of distantly related bacteria, significantly reduced the persister cell frequency upon ciprofloxacin challenge when added to exponentially growing and stationary phase *S. aureus* cells. Curiously, the persister reducing activity of *S. aureus* supernatants was also effective against persisters formed by either *S. carnosus* or *Listeria monocytogenes*. The persister reducing component, which resisted heat but not proteases and was produced in the late growth phase in an *agr* quorum-sensing dependent manner, was identified to be the phenol-soluble modulin (PSM) toxins. *S. aureus* express several PSMs, each with distinct cytolytic and antimicrobial properties; however, the persister reducing activity was specifically linked to synthesis of the PSMα family. Correspondingly, a high-persister phenotype of a PSMα mutant was observed upon fluoroquinolone or aminoglycoside challenge, demonstrating that the persister reducing activity of PSMs can be endogenously synthesized or extrinsically added. Given that PSMs have been associated with lytic activity against bacterial membranes we propose that PSM toxins increase the susceptibility of persister cells to killing by intracellularly acting antibiotics and that chronic and re-occurring infections with quorum sensing, *agr* negative mutants may be difficult to treat with antibiotics because of persister cells formed in the absence of PSM toxins.

## Introduction

Bacterial pathogens that are refractory to antibiotic treatment represent a serious threat to human health. Much attention has been paid to genetically defined antibiotic resistance mechanisms, while more recent awareness has been turned toward non-inherited or epigenetic resistance mechanisms as exemplified by the identification of intrinsic resistance genes (Morris et al., 2005; Vestergaard et al., 2016; El-Halfawy et al., 2017; Ito et al., 2017) or genes that upon induction provide tolerance phenotypes (Geisinger and Isberg, 2015; Haaber et al., 2015). Related to this category is the phenomenon of persisters. Persisters are formed when a fraction of a bacterial population enters a state of dormancy or slow growth that enables them to survive very high concentrations of antibiotics (Balaban et al., 2004; Lewis, 2010; Maisonneuve and Gerdes, 2014; Prax and Bertram, 2014; Schumacher et al., 2015). Clinically, persisters appear to be of critical importance as they contribute to persistent infections that are recurring and difficult to eradicate by antibiotics (Mulcahy et al., 2010; Conlon et al., 2015; Schumacher et al., 2015; Fisher et al., 2017; Van den Bergh et al., 2017). Strikingly, persisters can form a sub-population from which genetically encoded resistant variants emerge (Cohen et al., 2013; Levin-Reisman et al., 2017; Sebastian et al., 2017) and high-persister mutants are selected for by antibiotic exposure (Mechler et al., 2015; Van den Bergh et al., 2016). Consequently the formation of persister cells and methods to eradicate them is of great interest.

Persister cells appear to be formed stochastically during growth (termed type II persisters) (Balaban et al., 2004) and in response to adverse conditions. In *Escherichia coli* the stochastically induced persisters rely on the stringent response-mediated expression of guanosine tetraphosphate (ppGpp) and the concomitant cease in cell growth results in antibiotic tolerance (Amato et al., 2013; Maisonneuve et al., 2013; Verstraeten et al., 2015; Van den Bergh et al., 2016). Environmental conditions such as acid and oxidative stress, interactions with host environments and various fluctuations in metabolism or respiration also influences persister generation (Hong et al., 2012; Helaine et al., 2014; Orman and Brynildsen, 2015; Harms et al., 2016; Radzikowski et al., 2016). In stationary growth phase persister cells (termed type I persisters) generally form in a much greater fraction of the bacterial population than in exponential growth phase (Balaban et al., 2004; Keren et al., 2004; Lechner et al., 2012), and it has been speculated that biofilms may be largely composed of persister cells (Conlon et al., 2015; Waters et al., 2016). Entry into stationary growth phase is for many bacterial pathogens associated with induction of quorum sensing systems. The timing of quorum sensing activation is reflecting entry into stationary growth phase via sensing of so-called auto-inducers reaching a threshold concentration. Apart from that, quorum sensing systems are fundamentally different in Gram-negative and Gram-positive bacteria with the auto-inducing molecule generally being a peptide in the latter group. Quorum sensing coordinates diverse phenotypes such as expression of virulence factors, genetic competence, and biofilm formation (Miller and Bassler, 2001; Moreno-Gámez et al., 2017). Interestingly, quorum sensing induction itself has also been implicated in induction of antibiotic tolerance and persister cell generation in both Gram-negative and -positive bacteria (Möker et al., 2010; Leung and Lévesque, 2012; Que et al., 2013).

*Staphylococcus aureus* is a versatile pathogen causing a wide range of infections including abscesses, osteomyelitis, wound infections, endocarditis, and infections related to indwelling devices (Lowy, 1998; DeLeo et al., 2010). Chronic infections are also common and here persister cell formation appears to be central as exemplified by eradication of a chronic infection with an anti-persister compound (Conlon et al., 2013; Conlon, 2014). In contrast to other organisms the stringent response is not involved in *S. aureus* persister generation; however, the importance of the stationary growth phase for persister formation in *S. aureus* has been highlighted by the finding that genes normally expressed in stationary phase are induced in persister cells formed during exponential growth, and that these cells contain less ATP compared to growing cells again characterizing cells in the stationary growth phase (Conlon et al., 2016). Inspired by recent reports demonstrating that culture supernatants of *S. aureus* stimulate resuscitation of otherwise non-culturable *S. aureus* biofilm cells (Pasquaroli et al., 2013) and artificially generated *S. aureus* dormant cells (Pascoe et al., 2014), we set out to explore whether such spent medium would affect the frequency of persister cells observed in *S. aureus* cultures.

## Results and Discussion

### *S. aureus* Exponential Phase Persister Cell Frequency Is Reduced by Medium Spent by Staphylococci

Persister cell formation in *S. aureus* has previously been studied in response to lethal concentrations of ciprofloxacin or oxacillin using a variety of different strains (Keren et al., 2004; Lechner et al., 2012; Johnson and Levin, 2013; Conlon et al., 2016). Since some *S. aureus* strains are known to aggregate even at low cell densities and such aggregation confers antibiotic tolerance (Haaber et al., 2012), we chose to study formation of persister cells in exponential phase using strain Newman that reportedly is not or low-aggregating (Haaber et al., 2012). We conducted time-kill experiments by adding 20x MIC ciprofloxacin to growing cultures and observed the characteristic bi-phasic killing kinetics reflecting persister cell formation (Supplementary Figure S1) with 0.0001–0.001% surviving after 24 h, which is comparable to what was reported previously (Johnson and Levin, 2013). When repeating the time-kill protocol with persister cells collected at 24 h the killing kinetics and colony forming units (CFU) remained essentially unchanged demonstrating that the survivors are true persisters. In subsequent assays, we recorded persisters 24 h after antibiotic treatment.

**FIGURE 1:**
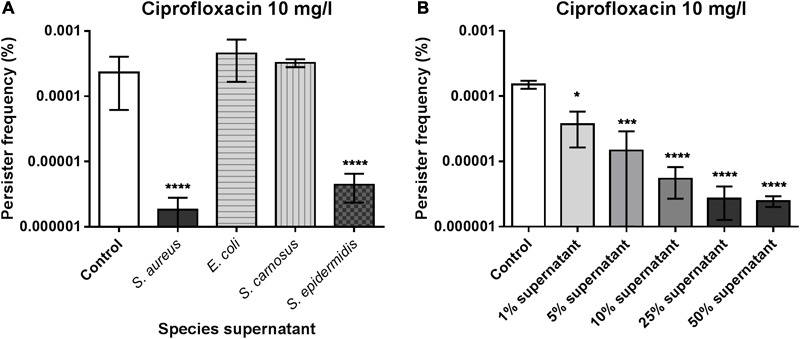
*Staphylococcus aureus* type II persister cell frequency is reduced in the presence of endogenous or closely related staphylococcal supernatants. *S. aureus* strain Newman was grown exponentially in the presence of 25% *S. aureus* (Newman), *E. coli, S. carnosus* or *S. epidermidis* stationary phase supernatants and challenged with 20x MIC of ciprofloxacin **(A)**. The persister cell frequency was determined as the fraction of survivors in individual cultures following 24 h antibiotic treatment. The dose-dependence of the *S. aureus* supernatant was assessed by 1–50% supplementation using 20x MIC of ciprofloxacin **(B)**. All experimental conditions were compared to the un-supplemented control (TSB medium). The data represent the mean persister frequencies ± SD calculated from three biological replicates (^∗^*P* < 0.05, ^∗∗∗^*P* < 0.001, ^∗∗∗∗^*P* < 0.0001).

When studying the influence of growth phases on persister formation in *S. aureus* strain Newman we discovered, by serendipity, that supplementing the TSB growth medium with 25% spent supernatant from stationary phase cells strikingly reduced exponential phase persister cells surviving lethal concentrations of ciprofloxacin by 10 to 100-fold when compared to cells just grown in plain TSB. Similar reduction was observed with supernatant obtained from the closely related species, *S. epidermidis*, while no effect was observed with supernatants from *S. carnosus* or *Escherichia coli* (**Figure 1A**). The supernatant effect is dose-dependent with moderate reduction in ciprofloxacin persisters observed already when supplemented with 1% supernatant while maximum activity, approaching the assay detection limit (1 CFU/ml), required 10–25% supernatant (**Figure 1B**). Importantly, the ciprofloxacin minimum inhibitory concentration (MIC) for *S. aureus* Newman (0.5 μg/ml) was unaffected by the presence of spent supernatant. We observed that the same supernatants affected the persister frequency also when selecting with oxacillin while the supernatant effect was negligible or absent when selecting for persisters with vancomycin (Supplementary Figure S2). To probe the generality of our observation we found that persister frequencies of two other *S. aureus* strains, SA564 and 8325-4, that are characterized as being able to form persister cells at low and relatively high frequencies, respectively, were also greatly affected by their respective supernatants (Supplementary Figure S3). We conclude that a component present in spent supernatants from *S. aureus* and closely related staphylococcal species significantly reduces persister cell formation and that the effect depends on the antibiotic with which they are isolated. Hence, we focused on persisters selected with ciprofloxacin onwards.

**FIGURE 2:**
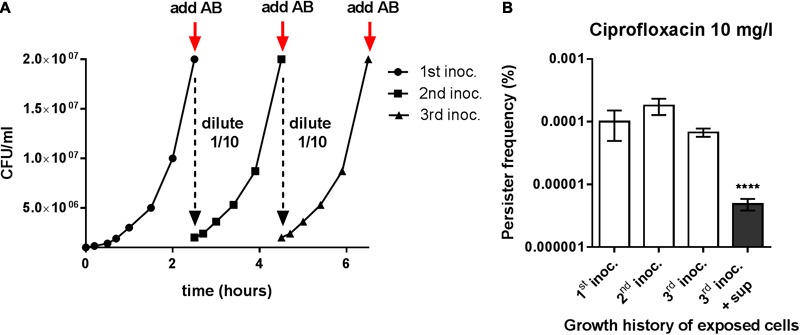
Persister reducing activity is unrelated to the stationary phase inoculum. **(A)** Setup of the re-inoculation experiment to assay for true type II persisters. The initial culture (1^st^ inoc.) was generated by a 1000-fold dilution of stationary phase cells in fresh TSB followed by growth for 2.5 h to enter exponential growth and reach a cell density of approximately 2 × 10^7^ CFU/ml. The subsequent cultures (2^nd^ and 3^rd^ inoc.) were derived from the initial culture by successive back-dilution (1/10) in fresh TSB and growth until reaching the same cell density. **(B)** Persister frequencies obtained after challenging the different *S. aureus* strain Newman cultures with 20x MIC of ciprofloxacin. The third culture was grown in the presence or absence of 25% stationary phase endogenous supernatant. The data represent the mean persister frequencies ± SD calculated from three biological replicates (^∗∗∗∗^*P* < 0.0001).

The persister cell frequency increases dramatically when *S. aureus* enters stationary phase (Keren et al., 2004). Therefore, we examined if persister cells formed in exponential phase are stationary phase cells carried over by dilution and if the effect of the spent supernatant was preserved when added to cells passaged for multiple growth cycles. By employing a successive back-dilution strategy (**Figure 2A**) we demonstrated that, in our assay, the persister cells formed during exponential growth are not carried over from the inoculum (**Figure 2B**) and that the spent supernatant remains equally active in reducing persister cells formation when added to cells after two rounds of passaging (**Figure 2B**). A previous study reported that *S. aureus* stationary phase supernatants are able to resuscitate dormant cells and that this effect could be mediated by two putative transglycosylases, IsaA and SceD (Pascoe et al., 2014). However, inactivation of either *isaA, sceD* or both genes did not alter the supernatant activity in our persister assay (Supplementary Figure S4), indicating that other factors are responsible for the observed effect.

### The Active Component Is Proteinaceous and Present within the >30 kDa Fraction

**FIGURE 3:**
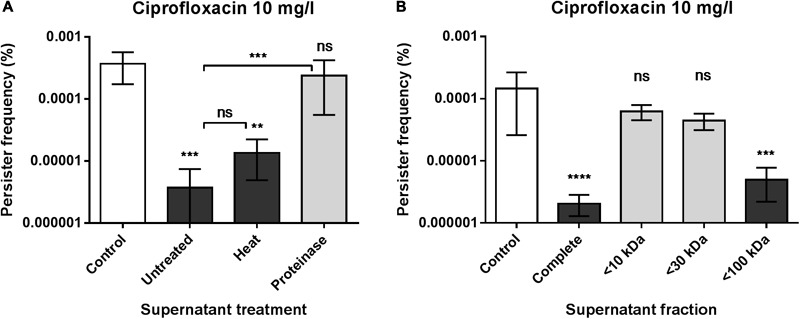
The active components is proteinaceous and present within the >30 kDa fraction. **(A)** Persister frequencies obtained from *S. aureus* strain Newman when grown in the presence of 25% untreated, heat- and proteinase-treated endogenous supernatant compared to the un-supplemented control. **(B)** Effect of supernatant fractions passed through 10, 30, and 100 kDa cut-off membranes on persister levels of *S. aureus* strain Newman. Comparison was made to the un-supplemented control and cultures added 25% of the complete endogenous supernatant. The data represent the mean persister frequencies ± SD calculated from three biological replicates treated with 20x MIC of ciprofloxacin (^∗∗^*P* < 0.01, ^∗∗∗^*P* < 0.001, ^∗∗∗∗^*P* < 0.0001).

To assess the nature of the component(s) responsible for the observed reduction in isolated persister cells, we subjected the supernatant to heat or protease treatment (**Figure 3A**) and spin-column fractionation (**Figure 3B**) prior to testing in the persister assay. By this, we defined the supernatant entity as being a heat-stable, proteinaceous substance that is enriched in the high molecular weight fraction; both of these characteristics further confirming that the active component is distinct from the resuscitation factor described by Pascoe et al. (2014) that was reported to be heat-sensitive and present in the <30 kDa fraction.

### The Active Supernatant Component Accumulates in Stationary Phase in an *agr*-Dependent Manner

**FIGURE 4:**
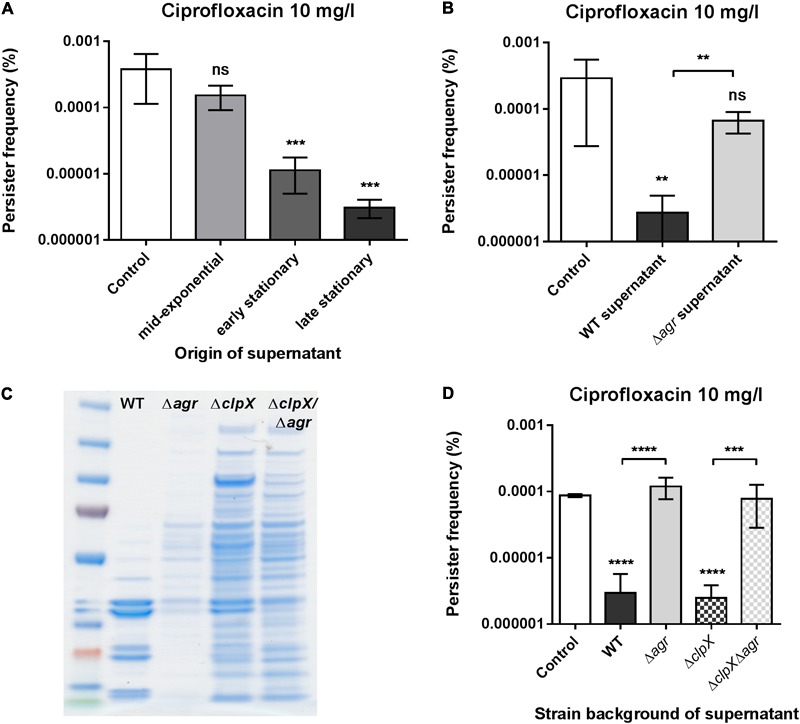
The supernatant activity is growth phase and *agr* quorum sensing dependent. **(A)** Persister frequencies obtained from *S. aureus* strain Newman supplemented with 25% endogenous supernatants obtained from different growth phases. **(B)** *S. aureus* strain Newman persister levels obtained when supplemented with 25% stationary phase endogenous supernatant from either wild type or NewmanΔ*agr* cells. **(C)** SDS-PAGE of total extracellular proteins present in stationary phase supernatants from *S. aureus* 8325-4 (WT) and its single (Δ*agr* and Δ*clpX*) mutant and double (Δ*agr*Δ*clpX*) mutant derivatives. Amount loaded corresponds to 20 μl of protein precipitate obtained from cultures of an equivalent of OD_600_ of 50. **(D)** *S. aureus* strain Newman persister levels obtained when supplemented with 25% stationary phase supernatants from either *S. aureus* 8325-4 (WT), its single (Δ*agr* and Δ*clpX*) mutants, or the double (Δ*agr*Δ*clpX*) mutant. All persister cells were selected using 20x MIC of ciprofloxacin and all supernatant treatments were compared to the un-supplemented control (TSB). The data represent the mean persister frequencies ± SD calculated from three biological replicates (^∗∗^*P* < 0.01, ^∗∗∗^*P* < 0.001, ^∗∗∗∗^*P* < 0.0001).

The central quorum sensing system of *S. aureus, agr*, is composed of a two component system with the membrane-associated AgrC histidine kinase and the response regulator AgrA that in response to auto-inducing peptides expressed by *agrB* and *agrD* induce expression of the effector molecule RNAIII (Novick, 2003). *S. carnosus* is considered non-pathogenic and strain TM300 is characterized by relatively low exoprotein production, likely a partial consequence of a mutation rendering the *agr* system non-functional (Rosenstein et al., 2009). This, together with the finding that the spent supernatants displayed persister repressing activity only if taken from post-exponentially growing cells (**Figure 4A**), suggested that the sought component(s) is under quorum sensing control. Indeed, we find that a supernatant taken from an *agr* quorum sensing deficient strain showed no modulatory activity on exponential phase persister cells selected with ciprofloxacin (**Figure 4B**), which we confirmed when obtaining the supernatant from another strain background (Supplementary Figure S5).

*agr* functionality has been associated with autolytic activity in *S. aureus* (Fujimoto and Bayles, 1998; Sakoulas et al., 2003) and has also been shown to facilitate release of cytoplasmic proteins, and cellular lipids, nucleic acids, etc. due to membrane damage (Ebner et al., 2017). To rule out that unspecific, spontaneous release of cytosolic proteins is a cause of activity we examined a *S. aureus clpX* mutant that has growth defects and is characterized by substantial release of cellular proteins into the supernatant (Frees et al., 2003; Bæk et al., 2016). This extraordinary release of cellular proteins was also evident in a *clpX*/*agr* double mutant (**Figure 4C**) and yet, loss of persister-reducing activity of the supernatant by *agr* dysfunction was equally evident in this background (**Figure 4D**) suggesting that the sought entity is directly regulated by *agr*.

**FIGURE 5:**
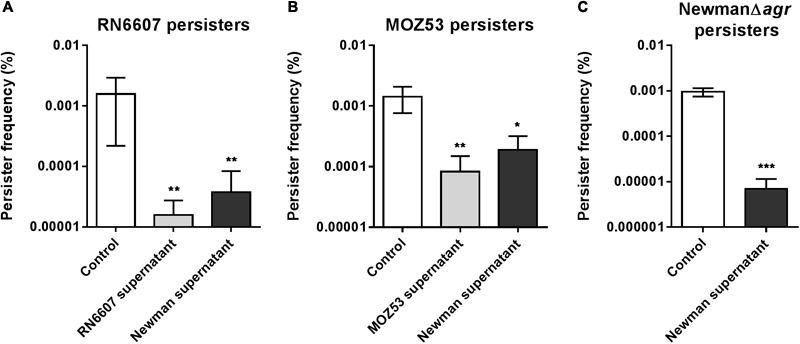
Persister reducing activity does not require induction of the *agr* response. Persister frequencies of *S. aureus* strains RN6607 (*agr* group II) **(A)** and MOZ53 (*agr* group III) **(B)** in the presence of 25% stationary phase endogenous supernatants or supernatant obtained from strain Newman (*agr* group I). **(C)** Effect of the wild type strain Newman stationary phase supernatant (25%) on persister cells isolated from strain NewmanΔ*agr*. Persister cells were isolated using 20x MIC of ciprofloxacin and all supernatant treatments were compared to the un-supplemented control (TSB). The data represent the mean persister frequencies ± SD calculated from three biological replicates (^∗^*P* < 0.05, ^∗∗^*P* < 0.01, ^∗∗∗^*P* < 0.001).

Induction of quorum sensing has previously been implicated in persister cell formation in other bacterial species (Möker et al., 2010; Leung and Lévesque, 2012). Although our fractionations had indicated the active factor to be of higher molecular weight, we examined if the small auto-inducing peptides secreted by *S. aureus* and present in the supernatants might prematurely activate the *agr* response and hereby influence the observed persister frequency. To test this, we employed the knowledge that a strain belonging to one *agr* specificity group does not activate the system of another group (Ji et al., 1997). The supernatant from strain Newman reduced the persister frequency of strains belonging to *agr* specificity groups II and III as did their endogenous supernatants (**Figures 5A,B**). As a final confirmation that our phenomenon does not rely on quorum sensing induction we found that the wild type supernatant significantly lowers the persister frequency also of an *agr* mutant (**Figure 5C**). Interestingly, we noted that the supernatant from *S. aureus* also negatively affects ciprofloxacin persister levels of *S. carnosus* and even *Listeria monocytogenes*, still in an *agr*-dependent manner (Supplementary Figure S6). This observation suggests that the supernatant affects persister cells via physiological means rather than some specific regulatory mechanism.

### An RNAIII Independent Activity – Persister Modulation via *agr*-Controlled Phenol-Soluble Modulins

**FIGURE 6:**
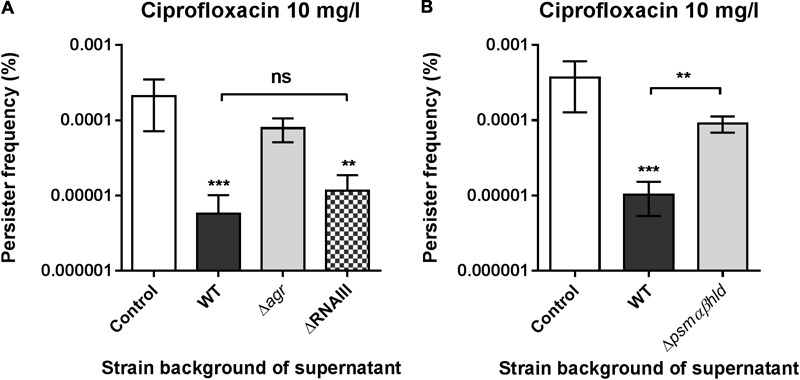
Phenol soluble modulins (PSMs) negatively affects exponential phase persister levels. **(A)** *S. aureus* strain Newman persister frequencies obtained when supplemented with 25% stationary phase endogenous supernatants from either strain Newman (WT), NewmanΔ*agr* (Δ*agr*) or NewmanΔRNAIII (ΔRNAIII). **(B)** Persister frequencies obtained when supplemented with 25% stationary phase supernatants from either strain LAC or LACΔ*psmαβhld*. Persister cells were selected using 20x MIC of ciprofloxacin and all supernatant treatments were compared to the un-supplemented control (TSB). The data represent the mean persister frequencies ± SD calculated from three biological replicates (^∗^*P* < 0.05, ^∗∗^*P* < 0.01, ^∗∗∗^*P* < 0.001).

The *agr* regulon is known to encompass a multitude of exoproteins and metabolic functions with the expression of the majority relying on the central regulatory molecule, RNAIII and a subset of targets being directly under the control of AgrA, the response regulator of the *agr* two-component system (Queck et al., 2008). When investigating which regulatory part of the *agr* regulon contributes to reduction of persister cells we found that the supernatant from RNAIII mutant cells remained equally effective as that of wild type cells in reducing the exponential phase persister frequency (**Figure 6A** and Supplementary Figure S7). Among the few genes under direct positive control by AgrA are those encoding the phenol-soluble modulins (PSMs) (Queck et al., 2008). They are a group of small amphipatic peptides expressed by *S. aureus* and other staphylococci divided into different classes with the α-class being ∼20 amino acids long and the β-class ∼44 amino acids as well as the δ-toxin that is encoded within RNAIII. In particular the α-class peptides are cytotoxic to many cell types, and work by non-specific membrane damage, while the β-class seems to lack cytotoxicity (Wang et al., 2007; Cheung et al., 2014). Interestingly, the PSMs are capable of associating into high molecular weight aggregates (Mehlin et al., 1999; Otto et al., 2004) and even form ordered amyloid structures that may stabilize biofilm structures (Schwartz et al., 2012). To further approach the hypothesis that the PSMs could be the factor influencing persister cell formation, we undertook supernatant fractionation by size-exclusion column chromatography. We found that while the active component(s) is indeed localized to the characteristic high molecular weight fractions of a wild type supernatant (i.e., they eluted early), and thus being consistent with the spin-column experiment (**Figure 3B**), a large proportion of the protein content within these fractions is in fact small peptides when analyzed by reducing SDS-PAGE (Supplementary Figure S8). Further, we observed that a supernatant withdrawn from a mutant unable to express PSM peptides (PSMα, PSMβ, and PSMδ families) had lost its ability to decrease the frequency of exponential phase persisters (**Figure 6B**) confirming that the PSMs are responsible for the reduction in exponential phase persister cell formation. Using synthetic PSMα3 we tried to recapitulate the anti-persister activity in a range of relevant concentrations without success (Supplementary Figure S9). It may be that the activity is related to one of the other peptides, a combination of several, or that the activity is confined to a processed form while at the same time being present as a high molecular weight entity.

### Phenol-Soluble Modulins Affect Stationary Phase Persisters

**FIGURE 7:**
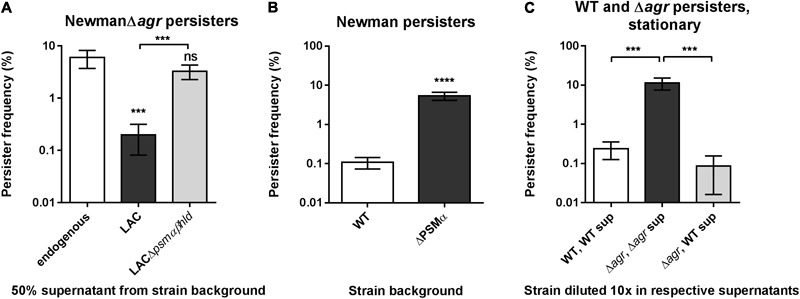
Phenol soluble modulins are strong determinants of *S. aureus* persister levels in stationary phase. **(A)** *S. aureus* strain NewmanΔ*agr* stationary phase persister frequencies obtained when supplemented with 50% stationary phase supernatants from either strain LAC or LACΔ*psmαβhld*. Persister cells were selected using 100x MIC of ciprofloxacin and treatments were compared to the control added endogenous supernatant only. **(B)** Stationary phase persister frequencies of *S. aureus* strain Newman and the derived *psmα* mutant selected with 100x MIC ciprofloxacin added directly to overnight cultures. **(C)** Stationary phase persisters of strain Newman (WT) and NewmanΔ*agr* (Δ*agr*) when resuspended in 90% endogenous supernatant prior to challenge with 100x MIC ciprofloxacin in comparison to persister level obtained with NewmanΔ*agr* in supernatant derived from WT. The data represent the mean persister frequencies ± SD calculated from three biological replicates (^∗∗∗^*P* < 0.001, ^∗∗∗∗^*P* < 0.0001).

Having pointed to a role of PSMs in reducing *S. aureus* exponential phase persister frequency when added exogenously we aimed to assess any relevance on cells that have entered stationary growth, a condition known to sharply increase the proportion of survivors of antibiotic treatment. First, we monitored stationary phase persisters when challenged with ciprofloxacin in the presence or absence of PSM-containing supernatant simultaneously. To eliminate the effect of endogenously expressed PSMs, we assessed persister cell formation in a Δ*agr* mutant of strain Newman. Upon addition of spent supernatant of either WT or PSM deficient cultures we observed a more than 10-fold reduction in stationary phase survivors in presence of PSMs (**Figure 7A**). Next, we asked if this finding translated into a direct difference in persister levels between a wild type and a *psm* mutant in stationary phase. Since the β-type PSMs have been reported to be expressed only in low amounts in *S. aureus in vitro* (Cheung et al., 2010), we focused on the α-type family. Certainly, a PSMα1-4 deficient derivative of *S. aureus* Newman displays well above 10-fold higher persister levels than wild type (**Figure 7B**) and we confirmed the PSMα-dependent phenotype by redoing the assay on specific PSMα and PSMβ mutant derivatives constructed elsewhere (Supplementary Figure S10A) (Tsompanidou et al., 2013). The generality of this paradigm was established by the significant effect obtained in reducing type I ciprofloxacin persisters of *S. aureus* strains SA564 and 8325-4 specifically by a PSMα-containing supernatant (Supplementary Figures S10B,C) and by confirming the high-persister phenotype of a specific *psmα* mutant constructed in SA564 (Supplementary Figure S11). Finally, correlating with the clear role of PSMs in reducing persister selection frequency, we note an apparent striking difference in persister frequency between wild type and *agr* mutant cells (compare **Figure 7A** and **7B**). We confirmed this notion in a direct comparison and that the 10 to 100-fold difference in persister levels between the two cell types is caused by the external environment as the high-persister phenotype of the *agr* mutant is negated by the wild type supernatant (**Figure 7C**).

## Conclusion

Here, we find that PSMα peptide toxins naturally produced by *S. aureus* and the related bacterium *S. epidermidis* are able to limit the *S. aureus* persister population. The effect is mediated both by endogenously produced peptides and by peptides added as part of spent staphylococcal supernatants, suggesting that they act exogenously. The high-persister phenotype of a stationary phase PSMα mutant upon fluoroquinolone treatment (**Figure 7B**) is mirrored when treating with the aminoglycoside gentamicin, but not upon challenge with wall-acting antibiotics, oxacillin and vancomycin, toward which a wild type cell remains fully tolerant also (Supplementary Figure S12). That the effect is confined only to some, yet unrelated, antibiotic classes suggests that the phenomenon is neither related to a direct killing of the persister subpopulation nor to a general resuscitation of persister cells. Further, we note that the persisters selected with oxacillin in exponential phase (Supplementary Figure S2B) were susceptible to the presence of supernatant. It may be that that effect is unrelated to PSMs or reflect a physiological difference between type I and type II persisters.

Recently, Xu et al. (2017) reported that inactivation of either *psmα* or *psmβ* genes increase persister formation over a time course of several days. They concluded that persister formation was regulated by an unclear mechanism and that the phenotype was specific to the fluoroquinolone levofloxacin. Here, we identified the activity of PSMs against persisters by a very different approach. When searching for a persister-reducing activity in spent staphylococcal supernatants we found the PSMα peptides to be responsible. The effect of the peptides was observed already within a 24 h antibiotic challenge; was evident on both exponential phase and stationary phase persisters and was seen when treated with intracellular antibiotics rather than only with fluoroquinolones. The extent to which our findings are consistent with those of Xu et al. (2017) warrants further study, including their observation that a deletion of either *psmα* or *psmβ* leads to a high-persister phenotype, which cannot be readily explained by a model that the peptides themselves reduces *S. aureus* persisters (i.e., PSMα is expected to be synthesized in the absence of *psmβ*). Importantly we observe that a PSM-containing supernatant limits the persister population not only of *S. aureus* but also of *S. carnosus* and the more distantly related *Listeria monocytogenes* (Supplementary Figure S6). Based on our findings we propose that PSMα peptides limit the persister population directly by interacting with the persister cells and increasing their susceptibility to antibiotics.

Originally PSMs were discovered as virulence factors that stimulate inflammatory responses and lyse human cells including leukocytes and erythrocytes with particularly the PSMα peptides being cytolytic (Wang et al., 2007; Cheung et al., 2014). However, activity against bacterial membranes have also been reported with the N-terminally processed PSMα1 and PSMα2 displaying antimicrobial activity against *Streptococcus pyogenes* (Joo et al., 2011). For *S. aureus*, PSMα peptides are found associated with the membrane where they are involved in release of lipoproteins and they promote colony spreading by exerting membrane surfactant activity (Tsompanidou et al., 2013; Kizaki et al., 2016). Strikingly, a recent report showed that particularly PSMα2 and PSMα3 can cause membrane damage in *S. aureus* to the extent that cellular components (proteins, nucleic acids, etc.) are released (Ebner et al., 2017). The toxicity of the PSM peptides against *S. aureus* explains previous observations of “non-classical” excretion of cellular proteins although the extent to which this influences viability is not clear (Ebner et al., 2017). Based on these observations we propose that the persister state of *S. aureus* cells is associated with changes in the bacterial membrane that reduce susceptibility to ciprofloxacin and gentamicin, however, in the presence of PSMα peptides, these changes are mitigated and susceptibility is restored. Intriguingly, Pader et al. (2016) unraveled a mechanism by which *S. aureus agr*-defective mutants specifically survives exposure to the membrane-active antibiotic daptomycin. While daptomycin is sequestered and inactivated by *S. aureus* by release of membrane phospholipids, this antibiotic-specific process is inhibited by PSMα excretion (Pader et al., 2016). It appears now that PSM-production by *S. aureus* may in fact alter the efficacy of multiple types of antibiotic exposure.

A surprising and interesting aspect of our findings is that they suggest that *S. aureus* limit the persister population under conditions where *agr* is induced and PSMs are expressed. Such conditions are found in acute infections where *agr* is considered to play an important role (Le and Otto, 2015). Persistent infections, however, are commonly reported with *agr* negative strains (Fowler et al., 2004; Park et al., 2013) and it appears that *S. aureus* can even diversify into acute and chronic subpopulations via what is termed *agr* bimodal switching within an otherwise clonal population (García-Betancur et al., 2017). In these cases, the PSM production will be absent and persisters are more likely to be present. Even short-term tolerance has been reported to be improved in strains lacking *agr* when exposed to lethal stressors such as ciprofloxacin or gentamicin (Kumar et al., 2017), however, it remains to be determined if these findings can be explained by lack of PSM production. In summary, we propose that *S. aureus* by producing PSMs limit the persister population under conditions of acute infection by increasing their susceptibility to antibiotics via membrane modulation whereas under chronic conditions the reduced PSM production allows the presence of persister cell with greatly reduced susceptibility to antibiotics.

## Materials and Methods

### Bacterial Strains and Growth Conditions

All strains used are listed in Supplementary Table S1. Strains were taken from frozen stocks (-80°C) and grown on tryptic soy agar (TSA, Oxoid) at 37°C. Liquid cultures were grown from single colonies in tryptic soy broth (TSB, Oxoid) at 37°C and were shaken at 200 rpm.

### Strain Constructions

The *isaA* and *sceD* mutations (Stapleton et al., 2007) were moved from the SH1000 background into strain Newman and the Δ*agr::tetM* mutation from RN6911 (Novick et al., 1993) was transferred into 8325-4; all by phage transduction (Φ11) and selection for integrated antibiotic resistance markers. Unmarked clean deletions of the *psmα1-4* locus were constructed in strain Newman and SA564 via temperature-sensitive allelic exchange using the shuttle vector pBASE6 (Geiger et al., 2012). Approximately, 1 kb chromosomal regions surrounding the *psmα* operon from the two strains were PCR amplified using primer pairs 5′-GATACAGAGCTCTTCCTGCATGCATAATTGCC-3′/5′-GAATTTTAAGTATTCAATTCGCTTAAATAAGATTACCTCCTTTGCTTATGAG-3′ and 5′-TTTAAGCGAATTGAATACTTAAAATTC-3′/5′-GATACAAGATCTCGAGTCAGCAGGATGGATC-3′, respectively. These products were subsequently joined in a spliced overlap extension PCR combining the forward and reverse primers from the previous reactions, thus generating strain specific deletion fragments that were cloned into pBASE6 via BglII/SacI. The resulting plasmids were purified from *E. coli* IM08B (Monk et al., 2015) and transformed directly into the wild type strains at 30°C followed by chromosomal integration by plating on TSA (10 μg/ml chloramphenicol) at 43°C. Plasmid cross-out was performed by passage at 30°C followed by plating on TSA (500 ng/ml anhydrotetracycline). Colonies were replica-plated to select for sensitivity toward chloramphenicol and successful allelic exchange were screened for by PCR amplification using primers 5′-TAAGACAACAAATTCTGAAGTAG-3′/5′-AGTTAGAATAACACCACCTGC-3′ positioned outside the chromosomal region used for homologous recombination.

### Preparation of Bacterial Supernatants

All supernatants were derived from TSB cultures grown from single colonies in shaking flasks at 37°C/200 rpm. Unless otherwise stated, supernatants were withdrawn after 24 h of growth. Cells were separated from the medium by centrifugation (10,000 *g* for 10 min) and the supernatant was passed through a 0.2 μm sterile filter (Minisart, Sartorius). Heat treated supernatant was prepared by heating in a water bath at 95°C for 20 min followed by an additional filtration step. Protease treatment was performed by addition of a *Streptomyces griseus* protease mixture (Sigma-Aldrich) at 1 mg/ml and incubation at 37°C for 30 min followed by heat inactivation (80°C for 20 min). Molecular weight fractionation was performed by passing a full supernatant through Vivaspin 6 ultrafiltration columns (Sartorius) with different molecular weight cut-offs (MWCOs) according to the manufacturer’s instruction. Size-exclusion chromatography was performed using an Äkta Pure 25 L1 and a Superdex 200 Increase 10/300 GL size exclusion column (both GE Healthcare Life Sciences). Supernatants were up-concentrated approximately 20-fold by use of Amicon^®^ Ultra 15 mL spin Filters (Millipore) with a 10 kDa cut-off and 500 μl of each sample were loaded onto the size-exclusion column and fractionated using 1x PBS as mobile phase and a flow-rate of 0.75 ml/min. All supernatants and fractions were freshly prepared or kept at -20°C until use.

### SDS-PAGE of Supernatant Proteins

Supernatant proteins were precipitated with ethanol (50% v/v) at 4°C overnight and resuspended in TE-buffer. Samples were analyzed on a NuPAGE^®^ Novex 4-12% Bis-Tris gel under reducing conditions using the XCell SureLock^®^ Mini-Cell system and stained with SimplyBlue^TM^ SafeStain according to the manufacturer (Thermo Fischer Scientific). Samples from the size-exclusion chromatography were mixed 1:1 with reducing (50 mM DTT) 2x Laemli buffer and run immediately using the same SDS-PAGE system.

### Determination of Minimum Inhibitory Concentrations

Minimum inhibitory concentrations of ciprofloxacin, gentamicin, oxacillin, and vancomycin (all Sigma-Aldrich) in TSB were estimated by standard twofold broth dilution. *S. aureus* strain Newman was inoculated in TSB (or TSB + 25% bacterial supernatant) and dispensed into microtiter trays added a twofold serial dilution of respective antibiotics reaching an initial cell count of approximately 5 × 10^5^ CFU/ml. Following 24 h of incubation at 37°C, MIC values were recorded as the lowest concentration of each antibiotic inhibiting visual growth.

### Persister Assay

The frequency of *S. aureus* persister cells in growing cultures (type II persisters) was determined by bactericidal antibiotic killing of the susceptible population and enumeration of surviving cells. Cells from an overnight culture was diluted 1/1000 in 2 ml fresh TSB and incubated in 15 ml test tubes at 37°C and 200 rpm for 2.5 h to reach exponential growth and a cell density of approximately 1–4 × 10^7^ CFU/ml. An aliquot was taken to allow determination of the actual CFUs (by serial dilutions on TSA plates) and the culture was supplemented with a lethal concentration (20x MIC) of antibiotic. After 24 h of incubation, 1 ml of culture was withdrawn and centrifuged (12,000 *g* for 5 min), washed with 0.9% NaCl to remove the antibiotic, and plated on TSA. Plates were incubated at 37°C for 24 h and the persister frequency was calculated as the plate count relative to the CFUs obtained at time 0 h. The persister level was arbitrarily set to 0.5 CFU/ml if a sample had less than 1 CFU/ml, i.e., below the detection limit. Stationary phase persister cells (type I persisters) were evaluated by transfer of 24 h cultures to new test tubes followed by addition of 100x MIC of antibiotic and comparing the CFUs before and after another 24 h of incubation. For some assays stationary phase cells were supplemented with exogenous supernatants prior to antibiotic challenge. A minimum of three biological replicates (overnight cultures originating from individual colonies) were included for each assay and experimental condition. All experimental results are representative of repeated independent experiments. Representative colonies from the persister assay were isolated to verify the non-inherited nature of persistence by confirmation of their susceptibility toward the antibiotic with which they were isolated and an unaltered persister frequency in subsequent assays.

### Statistical Analysis

Persister frequencies within individual experiments were compared by two-tailed Student’s *t*-test or One-way ANOVA followed by Dunnett’s or Tukey’s (where applicable) multiple comparisons test using GraphPad Prism version 6.00 for Windows, GraphPad Software, La Jolla, CA, United States^1^. Persister frequencies were log transformed prior to analysis to normalize the variance. Regular and multiplicity adjusted values of *P* < 0.05 were considered statistically significant.

## Author Contributions

MB and HI conceived the project and designed the work. MB and SL performed the experiments. MB, MV, and HI analyzed the data and drafted the manuscript. All authors approved the final version of the manuscript.

## Conflict of Interest Statement

The authors declare that the research was conducted in the absence of any commercial or financial relationships that could be construed as a potential conflict of interest.

## Footnotes

1 www.graphpad.com
