# Supplementary material for: Quorum Sensing-Regulated Phenol-Soluble Modulins Limit Persister Cell Populations in Staphylococcus aureus
Source: Front Microbiol. 2018 Feb 20;9:255. doi: 10.3389/fmicb.2018.00255 (PMC5826201; doi:10.3389/fmicb.2018.00255)
Supplement: Supplementary file 1 [file Table_1.pdf]

Quorum sensing-regulated phenol-soluble modulins limit persister cell populations in *Staphylococcus aureus*

Martin Saxtorph Bojer, Søren Lindemose, Martin Vestergaard and Hanne Ingmer

Supplementary data

Table S1.

**Strain** (*S. aureus* unless otherwise indicated)	**Characteristics**	**Source**
Newman	WT	Laboratory strain collection
Newman-agr	*agrA*::Tn551	(Paulander et al., 2012)
NewmanΔRNAIII	ΔRNAIII region::cat86	(Paulander et al., 2012)
NewmanΔ*sceD*	*sceD::kan*	This study
NewmanΔ*isaA*	*isaA::tet*	This study
NewmanΔ*sceD*Δ*isaA*	*sceD::kan/ isaA::tet*	This study
NewmanΔ*psmα*	Unmarked Δ*psmα1-4*	This study
NewmanΔ*psmα*	*psmα* deletion	(Tsompanidou et al., 2013)
NewmanΔ*psmβ*	*psmβ* deletion	(Tsompanidou et al., 2013)
NewmanΔ*psmαβ*	*psmα* and *psmβ* deletion	(Tsompanidou et al., 2013)
8325-4	WT	Laboratory strain collection
8325-4Δ*agr*	Δ*agr::tetM*	This study
WA400	ΔRNAIII region::cat86	(Janzon and Arvidson, 1990)
8325-4Δ*clpX*	In-frame deletion in *clpX*	(Frees et al., 2003)
8325-4 Δ*clpX*Δ*agr*	In-frame deletion in *clpX*/ Δ*agr::tetM*	(Frees et al., 2005)
SA564	WT	Laboratory strain collection
SA564Δ*psmα*	Unmarked Δ*psmα1-4*	This study
RN6607	WT, *agr* type II	Laboratory strain collection
MOZ53	WT, *agr* type III	Laboratory strain collection
JE2	WT	(Fey et al., 2013)
JE2-agrA	Transduced from NE1532 (Fey et al., 2013)	This study
JE2-agrC	Transduced from NE873 (Fey et al., 2013)	This study
E. coli MC4100	Common lab strain	Laboratory strain collection
S. carnosus TM300	Common type strain	Laboratory strain collection
S. epidermidis ATCC12228	Common type strain	Laboratory strain collection
LAC	WT	(Joo et al., 2011)
LAC*psmαβhld*	Complete *psm* knockout	(Joo et al., 2011)
L. monocytogenes EGD	Common type strain	Laboratory strain collection

Supplementary Fig 1.

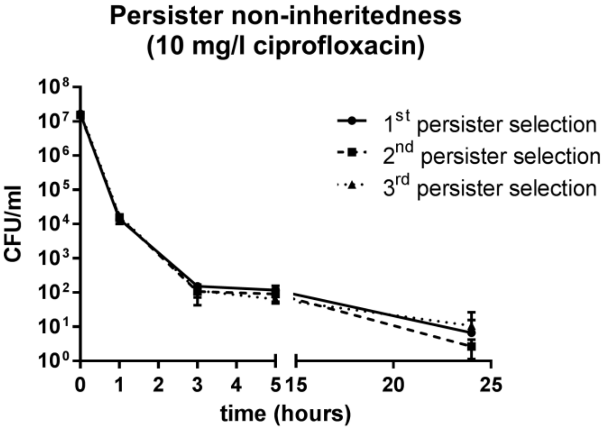

Supplementary Fig 1. Persister non-inheritedness. Time-kill experiment assaying survivors (CFU/ml) of exponential cultures of *S. aureus* strain Newman treated with 20xMIC ciprofloxacin. Each curve is based on biological triplicates (± SD) and cultures used for 2nd and 3rd persister selection were generated from colonies from survivors at the 24h time point of 1st and 2nd persister selection, respectively.

Supplementary Fig 2.

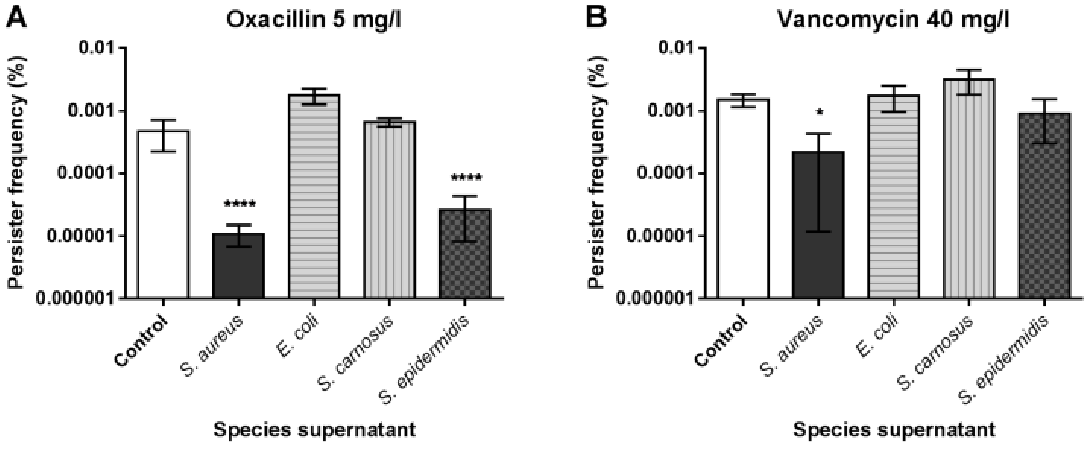

Supplementary Fig 2. Effect of stationary phase supernatant supplementation on exponential phase oxacillin and vancomycin persisters. *S. aureus* strain Newman was grown exponentially in the presence of 25% *S. aureus* (Newman), *E. coli*, *S. carnosus* or *S. epidermidis* stationary phase supernatants and challenged with 20xMIC of oxacillin (A) or vancomycin (B). The persister cell frequency was determined as the fraction of survivors in individual cultures following 24 h antibiotic treatment. Survival frequencies were compared to the un-supplemented control (TSB medium). The data represent the mean persister frequencies ± SD calculated from three biological replicates. (*$P<0.05$, ****$P<0.0001$).

Supplementary Fig 3.

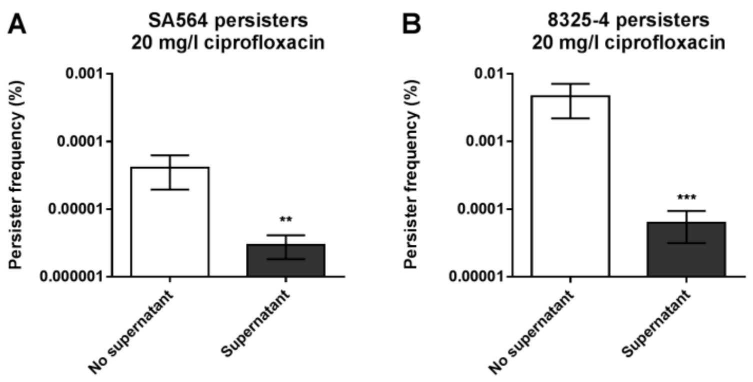

Supplementary Fig 3. Effect of endogenous supernatant supplementation on exponential phase persister frequency of *S. aureus* SA564 and 8325-4. *S. aureus* strains SA564 (A) or 8425-4 (B) were grown exponentially in the presence or absence of 25% of their own stationary phase supernatants and challenged with 20xMIC of ciprofloxacin. The persister cell frequency was determined as the fraction of survivors in individual cultures following 24 h antibiotic treatment and represented as the mean persister frequencies ± SD calculated from three biological replicates. (** *P*<0.01, ****P*<0.001).

Supplementary Fig 4.

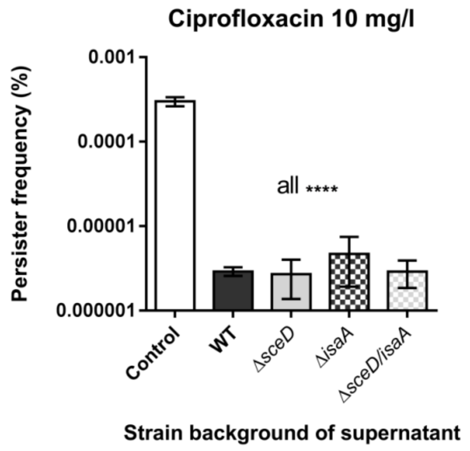

Supplementary Fig 4. No role of SceD or IsaA in supernatant activity. *S. aureus* strain Newman was grown exponentially in the presence of 25% of stationary phase endogenous supernatant from either wildtype, NewmanΔ*sceD*, NewmanΔ*isaA* or NewmanΔ*sceD*Δ*isaA* and challenged with 20xMIC of ciprofloxacin. The persister cell frequency was determined as the fraction of survivors in individual cultures following 24 h antibiotic treatment and represented as the mean persister frequencies ± SD calculated from three biological replicates. (*****P*<0.0001).

Supplementary Fig 5.

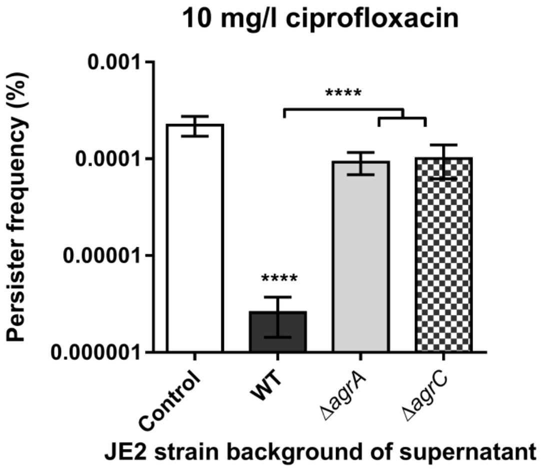

Supplementary Fig 5. Involvement of *agr* on supernatant activity of JE2. *S. aureus* strain Newman was grown exponentially in the presence of 25% of stationary phase supernatant from either JE2, JE2Δ*agrA* or JE2Δ*agrC* and challenged with 20xMIC of ciprofloxacin. The persister cell frequency was determined as the fraction of survivors in individual cultures following 24 h antibiotic treatment and represented as the mean persister frequencies ± SD calculated from three biological replicates. (*****P*<0.0001).

Supplementary Fig 6.

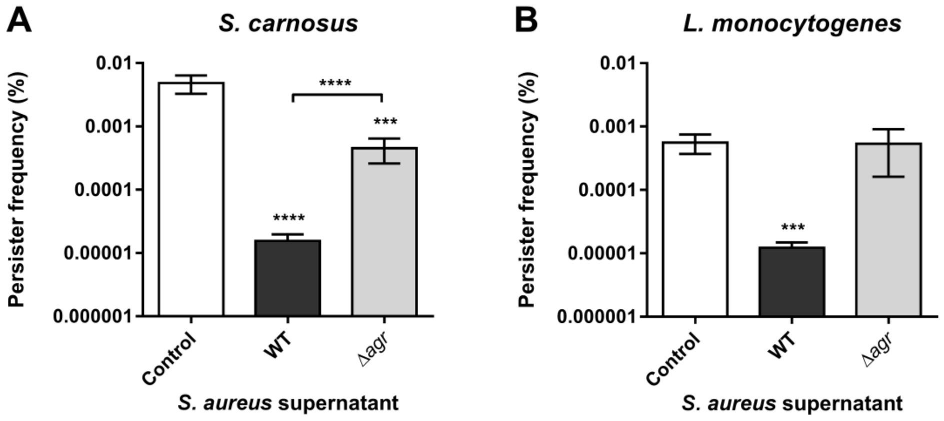

Supplementary Fig 6. Exponential phase persister frequencies of *S. carnosus* and *L. monocytogenes* are also affected by *S. aureus* supernatants in an *agr*-dependent manner. *S. carnosus* (A) or *L. monocytogenes* (B) were grown exponentially in the presence of 25% of stationary phase supernatant from either *S. aureus* strain Newman (WT) or NewmanΔ*agr* (Δ*agr*) and challenged with 20xMIC of ciprofloxacin. The persister cell frequency was determined as the fraction of survivors in individual cultures following 24 h antibiotic treatment in comparison with the un-supplemented control (TSB). The data represent the mean persister frequencies ± SD calculated from three biological replicates. (****P*<0.001, *****P*<0.0001).

Supplementary Fig 7.

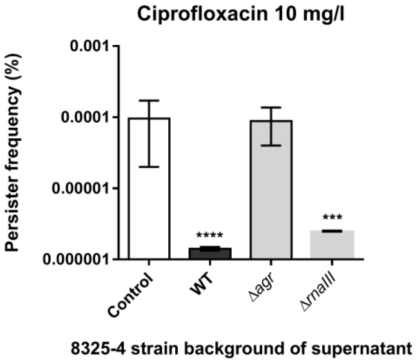

Supplementary Fig 7. Supernatant activity is independent of RNAIII also when derived from a strain 8325-4 background. *S. aureus* strain Newman persister frequencies obtained when supplemented with 25% stationary phase supernatants from either strain 8325-4 (WT), 8325-4Δ*agr* (Δ*agr*) or WA400 (ΔRNAIII). Persister cells were selected using 20xMIC of ciprofloxacin and all supernatant treatments were compared to the un-supplemented control (TSB). The data represent the mean persister frequencies ± SD calculated from three biological replicates. (****P*<0.001, *****P*<0.0001).

Supplementary Fig 8.

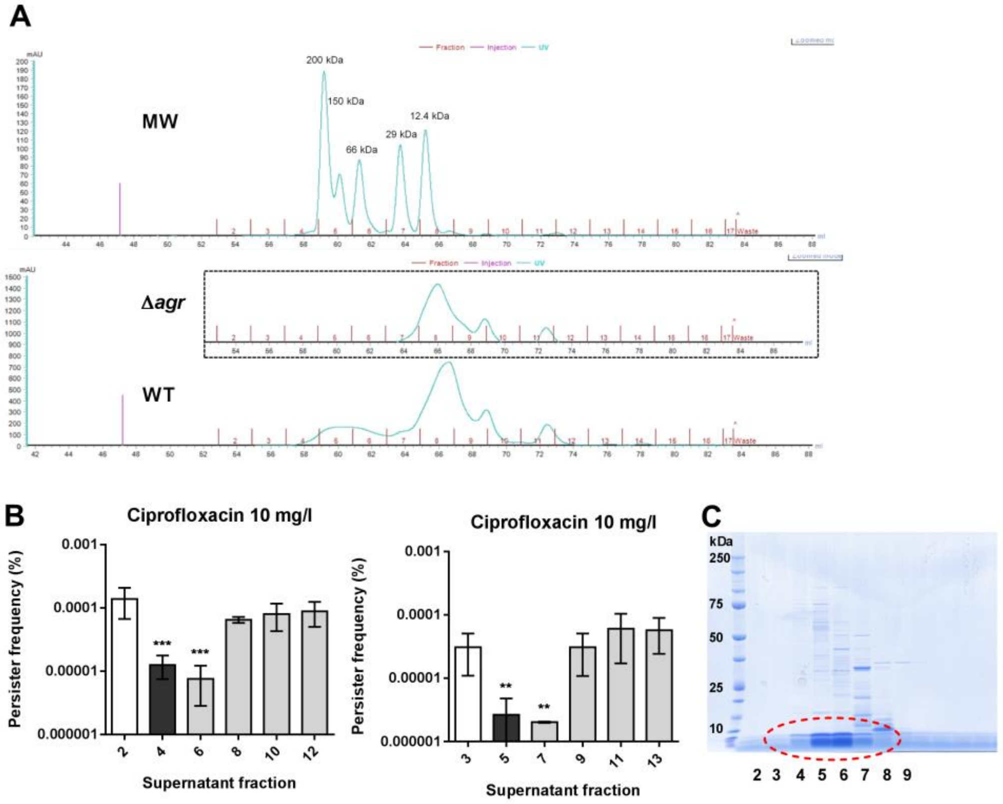

Supplementary Fig 8. Fractionation and activities. UV 280 nm absorbance diagrams from size-exclusion chromatography of either molecular weight standard (MW) or concentrated supernatants from *S. aureus* strain Newman (WT) or Newman Δ*agr* (Δ*agr*). The wild type supernatant gives rise to a characteristic peak of apparent high molecular weight proteins in fractions 4-7 (A). Fractions 4-7 in particular affect the exponential phase persister frequency of *S. aureus* strain Newman (***P*<0.01, ****P*<0.001) (B). The majority of the protein content in fractions 4-7 is composed by small (<5kb) peptides when analyzed by reducing SDS-PAGE (C).

Supplementary Fig 9.

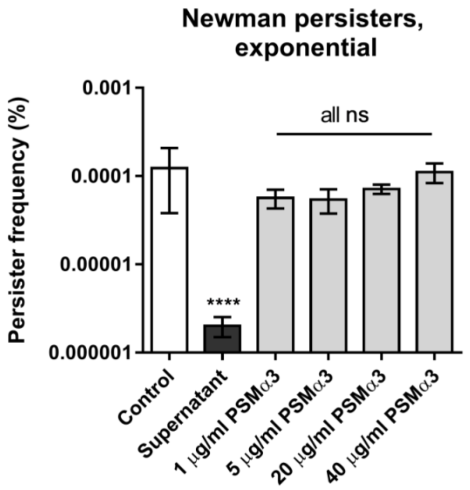

Supplementary Fig 9. *S. aureus* exponential phase persister frequency is not affected by synthetic PSMα3. *S. aureus* strain Newman was grown exponentially in the presence of either 25% stationary phase supernatant (as a positive control) or different concentrations of synthetic PSMα3 (N-formylated, >95% purity, EMC Microcollections, Germany). Persister cells were selected using 20xMIC of ciprofloxacin and all supernatant treatments were compared to the un-supplemented control (TSB). The data represent the mean persister frequencies ± SD calculated from three biological replicates. (*****P*<0.0001).

Supplementary Fig 10.

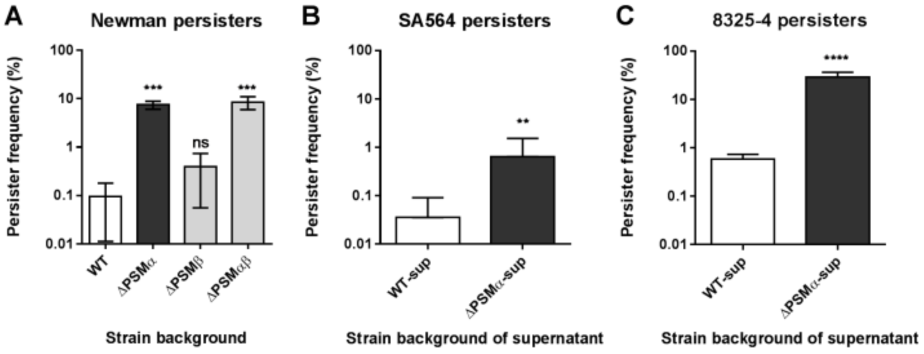

Supplementary Fig 10. Relevance of PSMα synthesis on stationary phase persister frequency. Stationary phase persister frequencies of *S. aureus* strain Newman and derived *psmα, psmβ,* and *psmαβ* mutants selected with 100xMIC ciprofloxacin added directly to overnight cultures (A). PSMα-containing supernatant negatively affects stationary phase persister levels in *S. aureus* strains SA564 (B) and 8325-4 (C) when diluting respective stationary phase cells 1/10 into Newman (WT) or NewmanΔPSMα (ΔPSMα) supernatants. Persister cells were selected using 100xMIC of ciprofloxacin and the data represent the mean persister frequencies ± SD calculated from three biological replicates. (***P*<0.01, ****P*<0.001, *****P*<0.0001).

Supplementary Fig 11.

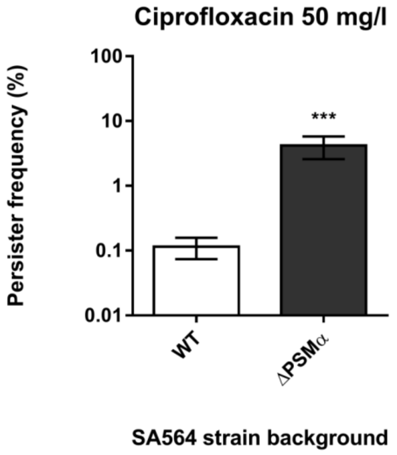

Supplementary Fig 11. PSMα deficiency leads to high stationary phase persister levels also in *S. aureus* strain SA564 Persister cells were selected using 100xMIC of ciprofloxacin added directly into stationary phase cultures of either SA564 (WT) or SA564ΔPSMα (ΔPSMα). The data represent the mean persister frequencies ± SD calculated from three biological replicates. (****P*<0.001).

Supplementary Fig 12.

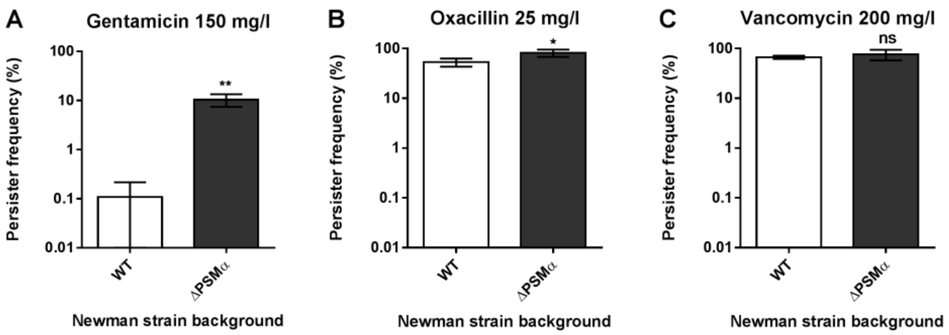

Supplementary Fig 12. PSMα proficiency leads to lowered stationary phase persister frequency also towards gentamicin, but not for oxacillin or vancomycin. Persister cells were selected by adding 100xMIC of gentamicin (A), oxacillin (B) or vancomycin (C) directly into stationary phase cultures of either Newman (WT) or NewmanΔPSMα (ΔPSMα). The data represent the mean persister frequencies ± SD calculated from three biological replicates. (**P*<0.05, ***P*<0.01).

References

Fey, P.D., Endres, J.L., Yajjala, V.K., Widhelm, T.J., Boissy, R.J., Bose, J.L., et al. (2013). A genetic resource for rapid and comprehensive phenotype screening of nonessential Staphylococcus aureus genes. *MBio* 4(1), e00537-00512.

Frees, D., Qazi, S.N., Hill, P.J., and Ingmer, H. (2003). Alternative roles of ClpX and ClpP in Staphylococcus aureus stress tolerance and virulence. *Molecular microbiology* 48(6), 1565-1578.

Frees, D., Sørensen, K., and Ingmer, H. (2005). Global virulence regulation in Staphylococcus aureus: pinpointing the roles of ClpP and ClpX in the sar/agr regulatory network. *Infection and immunity* 73(12), 8100-8108.

Janzon, L., and Arvidson, S. (1990). The role of the delta-lysin gene (hld) in the regulation of virulence genes by the accessory gene regulator (agr) in Staphylococcus aureus. *The EMBO journal* 9(5), 1391.

Joo, H.-S., Cheung, G.Y., and Otto, M. (2011). Antimicrobial activity of community-associated methicillin-resistant Staphylococcus aureus is caused by phenol-soluble modulin derivatives. *Journal of Biological Chemistry* 286(11), 8933-8940.

Paulander, W., Varming, A.N., Bæk, K.T., Haaber, J., Frees, D., and Ingmer, H. (2012). Antibiotic-mediated selection of quorum-sensing-negative Staphylococcus aureus. *MBio* 3(6), e00459-00412.

Tsompanidou, E., Denham, E.L., Becher, D., de Jong, A., Buist, G., van Oosten, M., et al. (2013). Distinct roles of phenol-soluble modulins in spreading of Staphylococcus aureus on wet surfaces. *Applied and environmental microbiology* 79(3), 886-895.
